# Combination of [^177^Lu]Lu-DOTA-TATE Targeted Radionuclide Therapy and Photothermal Therapy as a Promising Approach for Cancer Treatment: In Vivo Studies in a Human Xenograft Mouse Model

**DOI:** 10.3390/pharmaceutics14061284

**Published:** 2022-06-16

**Authors:** Marina Simón, Jesper Tranekjær Jørgensen, Harshvardhan A. Khare, Camilla Christensen, Carsten Haagen Nielsen, Andreas Kjaer

**Affiliations:** 1 Department of Clinical Physiology and Nuclear Medicine & Cluster for Molecular Imaging, Copenhagen University Hospital—Rigshospitalet & Department of Biomedical Sciences, University of Copenhagen, 2100 Copenhagen, Denmark; marina.simon@sund.ku.dk (M.S.); jtjoergensen@gmail.com (J.T.J.); h.khare@sund.ku.dk (H.A.K.); cch@minervaimaging.com (C.C.); chn@minervaimaging.com (C.H.N.); 2 Minerva Imaging, 3650 Ølstykke, Denmark

**Keywords:** photothermal therapy (PTT), nanoshells (NS), peptide receptor radionuclide therapy (PRRT), [^177^Lu]Lu-DOTA-TATE, somatostatin receptor (SSTR), cancer

## Abstract

Peptide receptor radionuclide therapy (PRRT) relies on α- and β-emitting radionuclides bound to a peptide that commonly targets somatostatin receptors (SSTRs) for the localized killing of tumors through ionizing radiation. A Lutetium-177 (^177^Lu)-based probe linked to the somatostatin analog octreotate ([^177^Lu]Lu-DOTA-TATE) is approved for the treatment of certain SSTR-expressing tumors and has been shown to improve survival. However, a limiting factor of PRRT is the potential toxicity derived from the high doses needed to kill the tumor. This could be circumvented by combining PRRT with other treatments for an enhanced anti-tumor effect. Photothermal therapy (PTT) relies on nanoparticle-induced hyperthermia for cancer treatment and could be a useful add-on to PRRT. Here, we investigate a strategy combining [^177^Lu]Lu-DOTA-TATE PRRT and nanoshell (NS)-based PTT for the treatment of SSTR-expressing small-cell lung tumors in mice. Our results showed that the combination treatment improved survival compared to PRRT alone, but only when PTT was performed one day after [^177^Lu]Lu-DOTA-TATE injection (one of the timepoints examined), showcasing the effect of treatment timing in relation to outcome. Furthermore, the combination treatment was well-tolerated in the mice. This indicates that strategies involving NS-based PTT as an add-on to PRRT could be promising and should be investigated further.

## 1. Introduction

In recent decades, the development of targeted therapies has been in the spotlight of research, in an effort to specifically treat diseases and tailor the treatment to the individual needs of the patients [1,2]. Peptide receptor radionuclide therapy (PRRT) has established itself as a safe, promising tool to fight neuroendocrine tumors (NETs) that overexpress somatostatin receptors (SSTRs), in particular SSTR2 [3,4]. These NETs are usually detected at late, metastatic stages when therapeutic options are limited and the prognosis is poor [5].

PRRT relies on α- and β- emitting radionuclides bound to a peptide targeting SSTR2 that are administered intravenously to the patient and travel towards the tumor [6]. Lutetium-177 (^177^Lu) is a β- and γ-emitting radionuclide used for PRRT, commonly labeled with a DOTA chelator to the somatostatin analogs octreotide ([^177^Lu]Lu-DOTA-TOC) or octreotate ([^177^Lu]Lu-DOTA-TATE). The latter was the first radiopharmaceutical for PRRT to be approved by the FDA in 2018 for the treatment of SSTR-positive gastroenteropancreatic NETs [7,8,9,10]. The β-particles emitted by ^177^Lu cause cytotoxicity in the cancer tissue with a maximal tissue penetration of 2 mm, killing cancer cells locally through DNA damage while minimizing damage to surrounding healthy tissues [11,12]. The γ-radiation allows for following the drug in vivo with molecular imaging techniques such as single-photon emission computed tomography (SPECT). Additionally, the half-life of 6.7 days provides ^177^Lu with a logistical advantage compared to many other radionuclides, because it allows for centralized manufacturing and distribution [13,14].

Although PRRT has shown positive results, the doses sometimes required to achieve a successful outcome (generally 7.4 GBq every 8 weeks for a total of 4 doses) can in turn trigger adverse effects, and achieving complete responses has proven challenging [15]. Hence, treatment strategies combining PRRT with other existing treatments that could potentially improve the outcome are currently being investigated in clinical and preclinical settings [16,17,18,19].

Photothermal therapy (PTT) utilizes light-absorbing molecules that accumulate in tumor tissue and cause severe hyperthermia when they are irradiated with near-infrared (NIR) light [20,21,22]. As a result, cancer cells are killed, and surrounding healthy tissue is spared. The most common agents used for PTT are gold nanoparticles, such as gold nanoshells (NS), due to their high photothermal efficiency, as well as their ability to accumulate in the tumor through the enhanced permeability and retention (EPR) effect [23,24,25,26]. Although PTT is a highly efficient, minimally invasive technique to induce local hyperthermia, it is usually insufficient to eliminate tumors when applied as a standalone treatment. This is primarily due to the limited light penetration depth of the NIR laser and heterogeneous accumulation of the photoabsorber in the tumor [21,22,27]. However, PTT has shown promise as an add-on to chemotherapy, radiotherapy, and surgery by improving drug accumulation, sensitizing cells to ionizing radiation, and removing tumor remnants after incomplete surgical resection [28,29,30,31,32,33]. Likewise, PTT could potentially improve treatment outcome when applied in combination with PRRT. On the one hand, it is likely that PTT-induced hyperthermia could sensitize cells to radiation damage. On the other hand, the radionuclide could reach some peripheral fraction of tumor cells not accessible by PTT [17].

In this study, the potential of applying a combinatorial strategy of [^177^Lu]Lu-DOTA-TATE PRRT and NS-based PTT was evaluated in mice bearing subcutaneous NCI-H69 (human small cell lung carcinoma) tumors.

## 2. Materials and Methods

### 2.1. Gold Nanoshells

The nanoparticles used were 800 nm Resonant Gold NS (BioPure™, lot JLF0015, NanoComposix Europe, Prague, Czech Republic). The NS have a gold shell (thickness of 16 nm) enclosing a silica core (diameter of 119 ± 5 nm). The surface is methoxy-polyethylene glycosylated (mPEGylated), and the total diameter is 150 ± 9 nm. The Au concentration is 0.96 mg/mL and the zeta potential is −34 mV, as reported by the supplier.

### 2.2. Flow Cytometry

NCI-H69 (human small cell lung carcinoma) suspension cells were cultured in RPMI 1640 medium (Thermo Fisher Scientific, Bleiswijk, The Netherlands) supplemented with l-glutamine, fetal bovine serum, and a penicillin/streptomycin mix. When cells reached appropriate confluence, they were harvested for flow cytometry to confirm SSTR2 expression. For this purpose, cells were washed first with PBS and after with flow cytometry staining buffer (0.5% BSA, 0.1% sodium azide, and 2 mM EDTA in PBS). Next, they were incubated with FC block to decrease unspecific staining before staining with mouse anti-human SSTR2 antibody (1:50, MAB4224, R&D Systems, Minneapolis, USA) and viability dye (1:400, eFluor™ 780, Thermo Fisher Scientific). This was followed by staining with a goat anti-mouse secondary antibody (1:400, Alexa Fluor^TM^ 594, Thermo Fisher Scientific). The controls were stained only with the viability dye or with the viability dye and the secondary antibody. Experiments were performed in triplicates. Samples were run in a BD LSRFortessa™ cell analyzer (BD Biosciences, Kongens Lyngby, Denmark) and the data was analyzed in the FlowJo software v.10.6 (FlowJo LLC, Ashland, OR, USA).

### 2.3. Animal Model

All animal experiments were approved by the Danish Animal Experimentation Council (2021-15-0201-01041) and undertaken in compliance with the directive 2010/63/EU of the EU legislation on the protection of animals used for scientific purposes.

The mice used for this study were female NMRI nude mice (five to six weeks of age), purchased from Janvier Labs (Genest St. Isle, France). Mice were allowed to acclimatize for a week upon arrival, with food and water *ad libitum*.

NCI-H69 cells were cultured as described previously and harvested for inoculation. For this procedure, the mice were anesthetized by breathing 4% sevoflurane, and ~3 × 10^6^ NCI-H69 cells in 100 μL (1:1 PBS: Matrigel) were injected subcutaneously into the left flank.

### 2.4. Histology

Mice bearing NCI-H69 tumors (*n* = 3) were euthanized, and tumors were taken out for histological analysis. First, tumors were fixated in paraformaldehyde followed by embedding in paraffin. Paraffin blocks of tissue were cut into 4 µm slices with a microtome (Rotary Microtome Microm HM355S, Thermo Fisher Scientific). After drying of slides, they were immersed in Histo-Clear solution to remove the paraffin and thereafter rehydrated in ethanol solutions. Before SSTR2 staining, slides were placed in a citrate buffer solution (pH = 6) to undergo microwave-based antigen retrieval. Tissue sections were then blocked with peroxidase solution (#S2023, Dako, Glostrup, Denmark) for 10 min and with bovine serum albumin solution for 20 min to block unspecific staining. Afterwards, slides were stained with an anti-SSTR2 antibody (1:1000, ab134152, Abcam, Cambridge, UK) for one hour at room temperature, followed by 40 min with an HRP-labelled polymer conjugated to a secondary antibody (anti-rabbit, EnVision + System-HRP Labelled Polymer, Dako). Following this, sections were incubated with DAB (Liquid DAB+ Substrate Chromogen System™, Dako) for eight minutes. Hematoxylin was used as counterstaining.

For hematoxylin and eosin (HE) staining, sections were stained with hematoxylin for five minutes and with eosin for two minutes. Sections were scanned on a Zeiss Axio Scan.Z1. Images were analyzed within the ZEISS Zen software (blue edition) (ZEISS, Jena, Germany).

### 2.5. [^64^Cu]Cu-DOTA-TATE PET/CT

[^64^Cu]Cu-DOTA-TATE was produced at DTU Nutech Hevesy Lab, Risø, Denmark, as previously described and approved under good manufacturing practice [34,35].

Mice bearing subcutaneous NCI-H69 tumors (*n* = 3) were injected with ~9 MBq of [^64^Cu]Cu-DOTA-TATE (~200 µL). They were anesthetized and scanned in a PET/CT scanner (Preclinical PET/CT Inveon, Siemens, Malvern, PA, USA) 1 h and 24 h after injection. During the scanning procedure, their temperature was kept stable using a heated bed. The settings for the CT scan were as follows: 360 projections, 65 kV voltage, 500 μA current, and 450 ms of exposure time. For the PET scans, the settings were: an energy window of 350–650 KeV and a timing window of 3.4 ns. Acquisition time was 5 min for the 1-h scan and 10 min for the 24-h scan. The maximum a posteriori algorithm was used to reconstruct the images, which were also CT attenuation corrected. PET and CT images were analyzed with the Inveon Research Workstation software (Siemens Preclinical solutions). Regions of interest (ROIs) were drawn on the tumor to quantify uptake as the percentage of injected dose per gram of tissue (% ID/g). A Gaussian filter (1.5 voxel size) was applied for smoother PET images post-quantification.

### 2.6. Autoradiography

After the 24-h [^64^Cu]Cu-DOTA-TATE PET/CT scan, the mice (*n* = 3) were euthanized. Tumors were excised and embedded in OCT before freezing in a cold 2-methylbutane solution. The tissue blocks were then cut with a cryostat (Leica CM 1860, Leica Biosystems, Brøndby, Denmark) in 20 μm slices. Three slices per mouse were then exposed to a phosphor screen (20 × 25 cm) overnight. Afterwards, the screen was scanned using an Amersham Typhoon Biomolecular Imager (GE Healthcare/Cytiva, Brøndby, Denmark) and the results were analyzed within the Image Quant TL 8.2 software (Cytiva).

### 2.7. Radiolabeling of [^177^Lu]Lu-DOTA-TATE

^177^LuCl_2_ (no-carrier-added, n.c.a, 0.4 M HCl, ITM, Kampen, The Netherlands) was added to DOTA-TATE in aqueous ammonium acetate (150 µL,0.25 M, pH 5.5) to a final specific activity of 20 MBq/µg. EtOH was added to the reaction to reach a final EtOH concentration of 5%. The mixture was left at 95 °C for 15 min. The mixture was purified on C8 SepPak light column, which was pre-activated with 5 mL 96% EtOH and 5 mL sterile filtered TraceSELECT water. The product was eluted with 0.5 mL of 96% EtOH and formulated with saline to reach a final activity concentration of 133.3 MBq/mL, peptide concentration of 6.7 µg/mL, and a final EtOH concentration of 5%.

The radiochemical purity at end-of-synthesis (EOS) was determined by radio-HPLC, analyzed on an XBridge C18 column (3.5 µM 4.6 × 50 mm) with flow of 1.5 mL/min. The HPLC mobile phase was solvent A, 0.1% TFA in H_2_O, and solvent B, 0.1% TFA in MeCN. Gradient: 0–7 min; 5–95% B; 7–8.5 min; 95–5% B; 8.5–11 min; 5% B. The retention time of [^177^Lu]Lu-DOTA-TATE was 2.7 min, and the specific activity was determined by UV absorption at 220 nm.

The amount of free ^177^Lu in the final product was evaluated by radio-thin-layer chromatography (radio-TLC) using an eluent 0.1 M citric acid of pH 5.4 on iTLC-SG plates, where the [^177^Lu]Lu-DOTA-TATE remains at the baseline, while ^177^Lu^2+^ ions elute with the solvent front.

### 2.8. SPECT/CT

Tumor-bearing mice were injected with ~20 MBq of [^177^Lu]Lu-DOTA-TATE (in 150 μL, corrected injected dose of 18.3 ± 0.1 MBq) and representative mice were scanned 1, 4, and 24 h after injection. Mice undergoing PTT were also scanned an additional time after laser treatment. Mice were anesthetized and placed in a nanoScan SPECT/CT scanner (Mediso Medical Imaging Systems, Budapest, Hungary). Their temperature was kept stable using a heated bed. The parameters for the CT scan were: 720 projections, 35 kVp of x-ray power, and 300 ms of exposure time. The frame time during the SPECT scan was set between 25 and 120 s, depending on the number of counts. The ^177^Lu photopeaks were: 208.40 keV (primary peak, 20% full width), 112.90 keV (secondary peak, 20% full width), and 56.10 keV (tertiary peak, 20% full width). SPECT images were reconstructed using Tera-Tomo™ 3D SPECT reconstruction software, and images were analyzed with the VivoQuant software (inviCRO, Boston, MA, USA).

### 2.9. Photothermal Treatment

Mice were injected with 190 µL of NS (5 × 10^10^ particles/mL) a day before PTT. For the laser treatment, they were placed under an 807-nm laser beam, under anesthesia (breathing sevoflurane 4%), and laying on their side so tumors were placed directly below the beam. Tumors were swabbed with glycerol to enhance light penetration [25]. The laser was turned on for five minutes and the intensity was tuned between 1.5–2 W/cm^2^ to reach and maintain the temperature within the desired range (~47–50 °C). The mice undergoing PTT without prior NS injection were used as a control for the unspecific heating of the laser at the highest intensity. Mice were given pain relief (buprenorphine) before PTT and twice more after six to eight hours.

### 2.10. Combination Treatment

Mice bearing ~150 mm^3^ NCI-H69 tumors were divided into seven groups (treatment detailed in Table 1); (1) Control group, (2) PRRT group, (3) PTT group, (4) PRRT + PTT day 1 group, (5) PRRT + laser day 1 group, (6) PRRT + PTT day 6 group, and (7) PRRT + laser day 6 group. Mice receiving PRRT were administered ~20 MBq in 150 µL on day 0. NS (190 μL, 5 × 10^10^ particles/mL) were injected a day before NS-based PTT. PTT was either performed on day 1 or day 6, and some mice received laser treatment without NS injection (laser groups). The PTT procedure was performed as described above. Tumor growth was monitored with a caliper and the tumor volume was determined with the formula: volume = ½ (length × width^2^). Mice were euthanized when tumors reached ~1500 mm^3^ (humane endpoint). A few mice were euthanized at a slightly smaller tumor size due to ulcerations on the tumor (also a humane endpoint).

### 2.11. Blood Analysis

Blood samples were taken from all mice at baseline, day 7, and day 14. This was done by restraining the animal and puncturing the sublingual vein with a needle. Approximately 100 µL of blood were collected in EDTA tubes and the hematology analysis was performed with the IDEXX ProCyte Dx* Hematology Analyzer.

### 2.12. Statistical Analysis

Maximum surface temperatures at the last time point (300 s) were compared with an unpaired t-test (all groups receiving NS-based PTT together as one group vs. all groups receiving laser treatment without NS). The survival data were used to create Kaplan–Meier survival curves and comparisons were made with the Log-rank (Mantel–Cox) test and corrected for multiple comparisons with the Benjamini–Hochberg procedure. Blood cell counts and weight measurements were compared with two-way ANOVA followed by a post hoc Tukey’s test. *p* values < 0.05 were considered statistically significant. All data are presented as mean ± standard error of the mean (SEM) and plotted on GraphPad Prism 9.

## 3. Results

### 3.1. Analysis of SSTR2 Expression in NCI-H69 Tumor Cells

**Figure 1:**
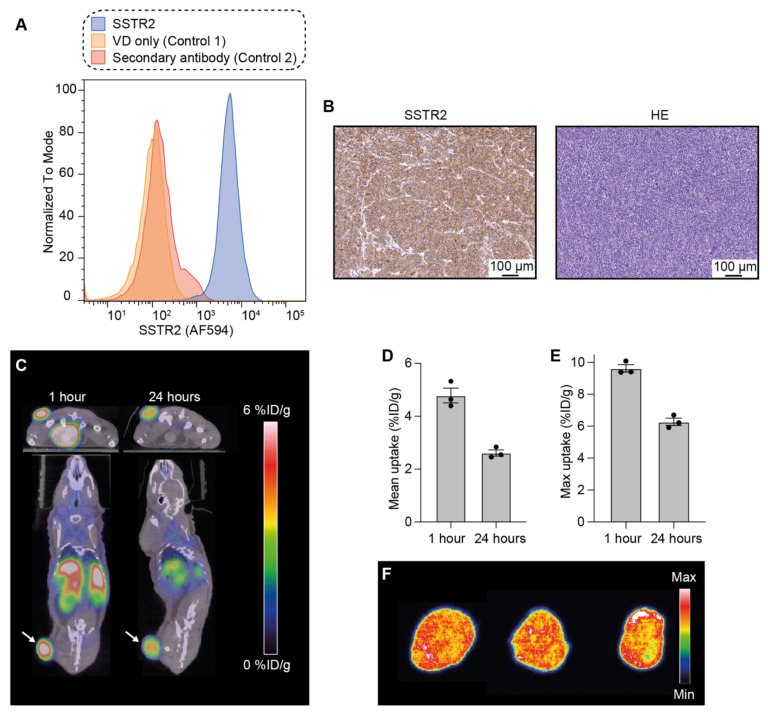
SSTR2 expression and [^64^Cu]Cu-DOTA-TATE tumor uptake in the NCI-H69 model. (**A**) Flow cytometric analysis of NCI-H69 cells to confirm SSTR2 expression (in triplicates). VD = viability dye. (**B**) Histological analysis to detect SSTR2 expression in NCI-H69 tumors and HE staining. (**C**) Representative PET/CT images of mice to observe [^64^Cu]Cu-DOTA-TATE distribution 1 h and 24 h after injection. Arrows point to the tumors. (**D**,**E**) Mean (**D**) and maximum (**E**) [^64^Cu]Cu-DOTA-TATE uptake in the tumor presented as percentage of injected dose per gram of tissue (% ID/g) 1 h and 24 h after injection. Data shown as mean ± standard error of the mean (SEM; *n* = 3). (**F**) Autoradiography of the [^64^Cu]Cu-DOTA-TATE tumor distribution (each tissue slice belongs to a different mouse).

First, the expression of SSTR2 was analyzed in NCI-H69 cells using flow cytometry with an anti-SSTR2 antibody. This confirmed the expression of SSTR2 in the cells compared to unstained controls (Figure 1A).

Thereafter, SSTR2 expression was also evaluated in tumor tissue. For this, NCI-H69 tumor-bearing mice were euthanized when tumors reached ~150–250 mm^3^, and the tumors were taken out for ex vivo tissue analysis. Tissue sections were stained with an anti-SSTR2 antibody and a high and homogeneous expression of the receptor was observed throughout the entire tumor tissue (Figure 1B). Hematoxylin and eosin (HE) analysis was also performed and showed the high cellularity of NCI-H69 tumors.

### 3.2. Uptake and Intratumoral Distribution of [64Cu]Cu-DOTA-TATE in Mice Bearing NCI-H69 Tumors

Next, the distribution of [^64^Cu]Cu-DOTA-TATE, a PET tracer for imaging of SSTR2-expressing tumors, was followed through PET/CT. This was performed in order to evaluate the uptake of DOTA-chelated octreotate in NCI-H69 tumors in vivo (Figure 1C).

NCI-H69 tumor-bearing mice (*n* = 3) were injected with ~9 MBq of [^64^Cu]Cu-DOTA-TATE and the animals were PET/CT-scanned 1 and 24 h post-injection.

We observed that one hour after injection, the mean uptake in the tumor was 4.8 ± 0.3 (mean ± standard error of the mean; SEM) percentage of the injected dose per gram of tissue (% ID/g, Figure 1D), and the maximum uptake was 9.6 ± 0.2% ID/g (Figure 1E). The mean uptake 24 h after injection decreased to 2.6 ± 0.2% ID/g; the maximum uptake was 6.3 ± 0.3% ID/g.

Additionally, to study the intratumoral distribution of [^64^Cu]Cu-DOTA-TATE, mice were euthanized after the last PET/CT scan, tumors excised, and sectioned into 20 μm tissue slices that were analyzed using autoradiography (Figure 1F). The signal from [^64^Cu]Cu-DOTA-TATE was homogeneous throughout the tumors, and thereby also in accordance with the uniform SSTR2 tumor expression observed through immunohistochemistry.

### 3.3. [^177^Lu]Lu-DOTA-TATE and Photothermal Therapy for the Treatment of SSTR2-Expressing Tumors In Vivo

After confirming the expression of SSTR2 and the tumor uptake of DOTA-TATE in our setup, we moved on to evaluating a combination therapy consisting of [^177^Lu]Lu-DOTA-TATE-assisted PRRT and NS-based PTT.

**Figure 2:**
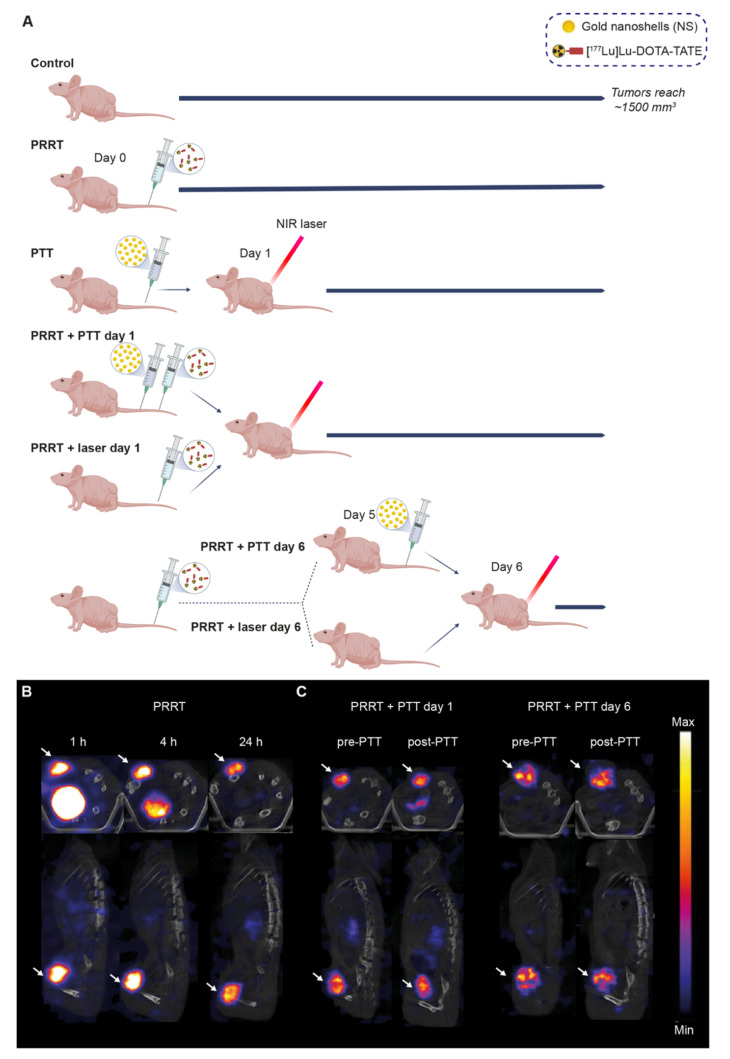
[^177^Lu]Lu-DOTA-TATE and NS-based PTT for the treatment of NCI-H69 tumors and SPECT/CT imaging. (**A**) Timeline for the treatment study for all groups. For the groups receiving PRRT, the [^177^Lu]Lu-DOTA-TATE injection was done on day 0. For the groups receiving PTT, the NS injection was done either on day 0 or day 5. NIR irradiation was performed a day after NS injection (day 1 or day 6). Tumor growth was followed with a caliper until endpoint (tumor volume of ~1500 mm^3^). (**B**) Representative SPECT/CT images from mice injected with [^177^Lu]Lu-DOTA-TATE (PRRT group) at 1, 4, and 24 h after injection. (**C**) SPECT/CT images from mice undergoing PRRT and PTT. Mice were scanned before and after PTT to evaluate the effect of therapy on tumor uptake. Arrows point to the tumors for both the axial and coronal images. The signal intensity is comparable at different timepoints in the same mouse but not between animals (*n* = 2 per group).

Mice bearing subcutaneous NCI-H69 tumors were divided into the following seven groups: (1) Control, (2) PRRT, (3) PTT, (4) PRRT + PTT day 1, (5) PRRT + laser day 1, (6) PRRT + PTT day 6, and (7) PRRT + laser day 6. The study timeline can be found in Figure 2A.

DOTA-TATE was successfully labeled with ^177^Lu with incorporation of 100% and a radiochemical yield of ≥99%. Radiochemical purity (RCP) was assessed by radio-HPLC and was determined ≥99%. The determination of free ^177^Lu in the final product was assessed by radio-TLC and no free ^177^Lu was found in the final formulated product. The specific activity of the final product was 20 MBq/µg. After the final injection, a sample of [^177^Lu]Lu-DOTA-TATE was reanalyzed on radio-HPLC and RCP was determined to be ≥94%.

In general, all mice receiving PRRT were injected intravenously with ~20 MBq of [^177^Lu]Lu-DOTA-TATE on day 0, a dose used in the literature to obtain a significant treatment effect but not a complete response [16]. SPECT/CT scans showed a specific tumor uptake of [^177^Lu]Lu-DOTA-TATE as early as one hour post-injection, and the tracer was quickly cleared from the rest of the body mostly through the kidneys (Figure 2B).

As for the PTT, mice were injected with the NS 24 h before being irradiated. Some mice received laser treatment without prior NS injection and were used as a control for the effect of heating induced by the laser (PRRT + laser groups).

In this study, mice undergoing combination therapy always received PTT after PRRT. This was established after a preliminary study showing that PTT performed immediately before injection of [^177^Lu]Lu-DOTA-TATE impaired tracer uptake, and consequently worsened the treatment effect (Figure S1). Therefore, we decided to perform PTT on day 1 or day 6 after [^177^Lu]Lu-DOTA-TATE administration. It was presumed that the effect of combining the therapies would be most significant when there was the highest possible level of ^177^Lu bound in the tumor. Day 1 was chosen as the earliest timepoint to facilitate the handling of the animals for PTT, as most of the unbound tracer would have been cleared at this timepoint (as observed on the SPECT/CT scan, Figure 2B). Day 6 was chosen since our preliminary study had also shown that tumors start shrinking around five to seven days after injection of [^177^Lu]Lu-DOTA-TATE (Figure S1). Thus, we hypothesized that PTT performed at this point could potentially improve the outcome. In addition, we observed that, at these timepoints, PTT did not seem to impair [^177^Lu]Lu-DOTA-TATE uptake in the tumor (Figure 2C).

**Figure 3:**
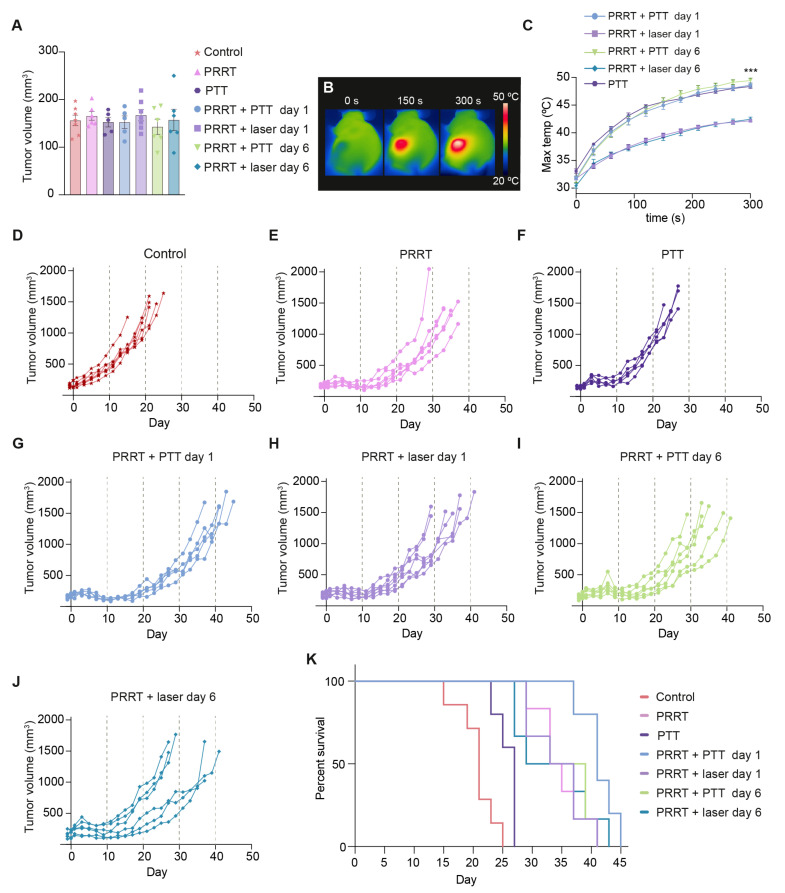
Evaluation of the treatment effect of [^177^Lu]Lu-DOTA-TATE PRRT and NS-based PTT in NCI-H69 tumor-bearing mice. (**A**) Tumor volume at day −1 for all groups. Data shown as mean ± SEM. (**B**) Representative thermal images of a mouse undergoing NS-based PTT. (**C**) Maximum surface temperatures on the tumors recorded with a thermal camera during the five-minute irradiation. Data shown as mean ± SEM. Temperatures at t = 300 s for all PTT mice vs. all laser mice were compared. *** represents *p* < 0.001. (**D**–**J**) Tumor growth curves for all mice in each of the groups. Animals were euthanized when tumors reached humane endpoints. (Control group; *n* = 7, PRRT group; *n* = 6, PTT group; *n* = 5, PRRT + PTT day 1 group; *n* = 5, PRRT + laser day 1 group; *n* = 7, PRRT + PTT day 6 group; *n* = 6, and PRRT + laser day 6 group; *n* = 6). (**K**) Survival curves for all groups.

The mean tumor size for all groups (~150 mm^3^) on randomization day (day −1) is reported in Figure 3A. All mice treated with NS-based PTT (PRRT + PTT day 1, PRRT + PTT day 6, and PTT groups) reached very high surface temperatures after five minutes of irradiation (overall mean of 48.8 ± 0.2 °C), detected with a thermal camera (Figure 3B,C). These temperatures were comparable to what has previously been reported for NS-based PTT in other tumor models [33,36,37], and significantly higher (*p* < 0.001) than the ones reached on the tumors of the animals that were not injected with NS but were still treated with the laser (PRRT + laser day 1 and PRRT + laser day 6 groups, mean of 42.3 ± 0.2 °C).

As expected, all groups receiving [^177^Lu]Lu-DOTA-TATE experienced a slowdown in tumor growth and an increase in survival (*p* < 0.05) compared to both PTT and Control groups (Figure 3D–K). PTT as a monotherapy also led to improved survival when compared to the control mice (*p* = 0.02), but the treatment was not as effective as PRRT.

As for the combination treatment, we saw an improved response when performing NS-based PTT one day after PRRT, with tumors growing at a slower pace and an elongated survival (PRRT + PTT day 1 vs. PRRT; *p* = 0.03). Interestingly, this effect was not observed when PTT was performed at a later timepoint after [^177^Lu]Lu-DOTA-TATE injection (PRRT + PTT day 6 group vs. PRRT; *p* = 0.81).

The groups receiving [^177^Lu]Lu-DOTA-TATE and laser treatment without NS (PRRT + laser day 1 and PRRT + laser day 6 groups) responded in a similar way to the group of mice exclusively receiving [^177^Lu]Lu-DOTA-TATE (PRRT group). This showed that the laser alone, as expected, did not trigger an improvement in treatment outcome.

Median survival was 41 days for the PRRT + PTT day 1 group, 36 days for the PRRT + PTT day 6 group, 35 days for the PRRT + laser day 1 group, 34 days for the PRRT group, 33 days for the PRRT + laser day 6 group, 27 days for the PTT group and 21 days for the Control group (Figure 3K).

### 3.4. The Combination of [^177^Lu]Lu-DOTA-TATE and Photothermal Therapy Is Well-Tolerated in Tumor-Bearing Mice

**Figure 4:**
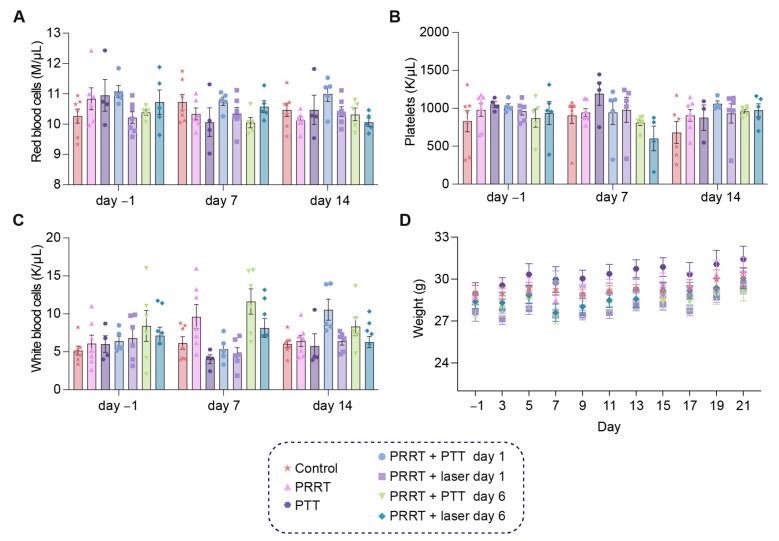
Blood cell analysis after combined [^177^Lu]Lu-DOTA-TATE and PTT. (**A**) Red blood cells (M/μL) at day −1, day 7, and day 14 for all groups (Control, PRRT, PTT, PRRT + PTT day 1, PRRT + laser day 1, PRRT + PTT day 6, and PRRT + PTT day 6). (**B**) Platelets (K/μL). (**C**) White blood cells (K/μL). (**D**) Weight (g) measured every other day for all groups, and plotted until day 21 to ensure sufficient representation of mice from all groups. Data shown as mean ± SEM. (*n* = 4–7 per group).

We also wanted to study the possible hematological toxicity derived from the therapy, since PRRT is known to have a negative effect on some blood components. For this, we took sublingual blood samples one day before treatment (day-1), as well as 7 and 14 days after [^177^Lu]Lu-DOTA-TATE administration and analyzed changes in red blood cells, platelets, and white blood cells (Figure 4A–C). Weight was also measured every other day (Figure 4D). Although variability within groups could be observed for some of the measurements (particularly for the white blood cell analysis), from the data we concluded that our treatment conditions did not lead to any critical hematological toxicity at the timepoints analyzed, and red blood cells, platelets, and white blood cells remained at similar values before and after therapy. The combinatorial strategy was well-tolerated by the mice, also confirmed by the stable weight throughout time for all groups.

## 4. Discussion

PRRT is a valuable technique for the localized killing of tumors through internal radiation. It relies on α- or β-emitting radionuclides bound to a tumor-targeting peptide that is injected systemically and travels towards the tumor, where it binds and delivers ionizing radiation locally to the cancer cells. The radiolabeled somatostatin analog [^177^Lu]Lu-DOTA-TATE has proven valuable for the treatment of SSTR2-expressing neuroendocrine cancers [8,10]. Nonetheless, the use of PRRT also has some limitations. If the tumor-to-background ratio is insufficient, high doses of the radionuclide need to be administered to achieve treatment effect, and consequently, high radiation doses are also delivered to healthy tissue [38]. Because of this, only patients with sufficiently high tumor uptake relative to the liver (Krenning scale) are eligible for PRRT [39]. The healthy tissues that can be affected by the therapy are determined by the blood circulation time, biodistribution, and excretion route of the probe. In patients treated with [^177^Lu]Lu-DOTA-TATE, hematological and nephrotoxicity are the most common findings [40]. Peptide-based tracers are primarily cleared by glomerular filtration, resulting in high doses of radiation being absorbed by the kidneys. The nephrotoxicity can somewhat be reduced by amino-acid infusion, which slows down the filtration rate of the peptide and decreases the radiation dose. Nonetheless, the kidneys together with the bone marrow, very sensitive to radiation, become the dose-limiting organs [41]. It is necessary to reduce the dose of the radionuclide to limit radiotoxicity; however, this can also hamper treatment effect [42]. One way to overcome some of these limitations is to use PRRT in combination with other existing treatments. For example, [^177^Lu]Lu-DOTA-TATE-based PRRT has been tested in combination with other drugs such as platinum chemotherapy and immune checkpoint inhibitors [2,19,43]. Moreover, [^177^Lu]Lu-DOTA-TATE therapy has also been able to reduce tumor size to operable levels by acting as a useful neoadjuvant treatment [44]. Overall, the approach of combining PRRT with other treatment modalities looks promising, yet more knowledge is still needed on how to apply the treatment in combination with other therapies, to achieve a synergistic effect while minimizing adverse effects.

PTT is a minimally invasive technique that causes tumor cell killing through localized hyperthermia [20,45]. However, PTT as a monotherapy presents certain limitations, such as passive and heterogeneous delivery of light-absorbing nanoparticles to the tumor and restricted NIR laser light penetration, which is why preclinical focus has been shifted to applying the therapy as an add-on to improve other established cancer treatments [22,31,46].

In this study, we hypothesized that a combination of PRRT and PTT could improve treatment outcome in a mouse cancer model. We therefore tested a combination strategy where a ~20 MBq [^177^Lu]Lu-DOTA-TATE injection was followed by NS-based PTT, performed either one day or six days after PRRT. We found that mice that underwent the combination treatment with PTT performed one day after [^177^Lu]Lu-DOTA-TATE injection (PRRT + PTT day 1 group) presented a slowdown in tumor growth and longer survival compared to all the other groups. Interestingly, when PTT was performed at a later timepoint, it did not improve the outcome. Thus, this study also highlighted the importance that the timing of the treatments can have on the outcome.

It is known that hyperthermia can increase blood flow and extravasation from tumor vessels [47,48]. This improves the delivery of many therapeutics to the tumor, thereby enhancing their effect. Moreover, hyperthermia can also potentiate the effect of radiation therapy by improving tumor oxygenation and impairing DNA repair [49,50,51,52]. Different treatment schedules have been used, but generally, a better outcome is observed when cancer cells are exposed to hyperthermia and radiation at the same time or as close to each other as possible [50]. As the interval between the two treatments increases, the radiosensitization generated by the high temperatures decreases. However, using our setup in a preliminary study, we experienced that when PTT was performed immediately before PRRT administration, it led to lower [^177^Lu]Lu-DOTA-TATE tumor uptake compared to animals that were exclusively treated with PRRT. This also resulted in worse treatment outcomes (Figure S1). The reason for this is most likely related to the fact that [^177^Lu]Lu-DOTA-TATE reaches the tumor by active targeting, i.e., by binding to SSTR2. But the prior photothermal ablation resulted in tumor death and a reduction of the target, which led to decreased tracer binding and hindered treatment outcome.

Based on these results, PTT was then administered after [^177^Lu]Lu-DOTA-TATE injection, and we chose to perform PTT one day or six days post-injection. On day 1, high levels of [^177^Lu]Lu-DOTA-TATE were present in the tumor, and the interval between the two treatments was kept as short as possible to allow for a potential synergistic effect. Day 6 was selected because tumors start to shrink after PRRT around this timepoint (Figure S1), and we reasoned that PTT performed then might be able to eradicate or radiosensitize tumor cells that did not initially respond to PRRT. In addition, at these timepoints, PTT did not seem to have an effect on the tumor uptake of [^177^Lu]Lu-DOTA-TATE (Figure 2C).

However, the combination strategy only led to an improvement in survival when it was performed one day after [^177^Lu]Lu-DOTA-TATE injection and not at the later timepoint (Figure 3K). This can likely be explained by the well-established knowledge that hyperthermia and radiation should be performed as close to each other as possible to obtain the synergistic effect [50]. After [^177^Lu]Lu-DOTA-TATE has distributed and accumulated in the tumor, PTT will result in ablation of a fraction of tumor cells, while other cells could at the same time be sensitized to radiation. The reason this effect is not seen when PTT is performed at the later timepoint could be down to lower levels of ^177^Lu being present in the tumor on day 6. However, differences in uptake and intratumoral distribution of NS after a longer period of PRRT might also affect the outcome of the combinatorial strategy and should be considered.

Recently, structures that combine α- or β-emitting radionuclides with gold nanoparticles for joint PRRT and PTT have been developed [17,53,54]. These two-in-one particles make treatment design more straightforward, and hyperthermia can potentially be induced in the same location as radiation. However, their action is limited to the areas of the tumor where the particle accumulates. Alternatively, dividing radiation and PTT by administering two agents (i.e., a photothermal gold nanoparticle and a radionuclide) results in two different spatial distributions in the tumor. This could potentially result in the treatment reaching a larger fraction of the cancer cells. However, there are still multiple factors to consider when applying a combination strategy of PTT and radionuclide therapy, and more studies are needed to develop the application.

Since PRRT can trigger toxicity, the effect of the therapy on blood cells was also studied, and we analyzed relevant cell populations by taking blood samples at different timepoints.

We observed that the therapy was well-tolerated, and we did not detect any myelosuppression or significant weight change in the mice over time. This could also be related to the low dose of ~20 MBq ^177^Lu. This dose worked well for our study, but could potentially be increased, leading to a more successful outcome while still limiting toxicity. Moreover, in clinical settings, dose administration is done in fractions, which helps increase the dose delivered to the tumor while minimizing side effects [55].

Finally, although it makes it extremely useful for our purpose, it is a limitation of this study that the high and homogenous SSTR2 expression in the NCI-H69 tumor model cannot be fully interpolated to human tumors. Cell-line derived tumor models rely on single identical clones, but this does not fully match the situation in human tumors, composed of several different cell genotypes and phenotypes. These can also present highly variable levels of SSTR2 expression [56]. Therefore, performing PRRT targeting SSTR2-expressing tumors in humans is not as straightforward as in mouse models. Nevertheless, our results represent a first positive proof-of-concept for combined PRRT and NS-based PTT.

## 5. Conclusions

In conclusion, we performed a combination strategy involving two methods that have already been applied in humans as monotherapies, [^177^Lu]Lu-DOTA-TATE-based PRRT and NS-based PTT, which led to improved treatment outcome with no obvious toxicity in NCI-H69 tumor-bearing mice. However, the timing of applying PTT seems crucial for the success of the approach. Our studies show the potential of PTT as an add-on to [^177^Lu]Lu-DOTA-TATE-based PRRT, and this could possibly also be extrapolated to other types of radionuclide therapy.

## Figures and Tables

**Table 1:** Experimental treatment groups. Mice were divided into seven groups, receiving different treatments. For PRRT, all animals were administered ~20 MBq of [^177^Lu]Lu-DOTA-TATE on day 0. For PTT, all animals were injected with NS 24 h prior to laser irradiation (day 1 or day 6). PTT control animals (laser groups) were irradiated without previously receiving NS.

Group	Treatment	*n*
Control	No treatment	7
PRRT	[^177^Lu]Lu-DOTA-TATE injection (day 0)	6
PTT	NS-based PTT (day 1)	5
PRRT + PTT day 1	[^177^Lu]Lu-DOTA-TATE injection (day 0) and NS-based PTT (day 1)	5
PRRT + laser day 1	[^177^Lu]Lu-DOTA-TATE injection (day 0) and laser treatment (without NS, day 1)	7
PRRT + PTT day 6	[^177^Lu]Lu-DOTA-TATE injection (day 0) and NS-based PTT (day 6)	6
PRRT + laser day 6	[^177^Lu]Lu-DOTA-TATE injection (day 0) and laser treatment (without NS, day 6)	6

## Data Availability

The raw data are available upon request.

## Supplementary Materials

The following supporting information can be downloaded at: https://www.mdpi.com/article/10.3390/pharmaceutics14061284/s1, Figure S1: PTT followed by PRRT for the treatment of NCI-H69 tumor-bearing mice. (A) Study timeline. The control group (*n* = 5) did not receive any treatment. The PRRT group (*n* = 7) was injected with ~30 MBq of [^177^Lu]Lu-DOTA-TATE on day 0. The PTT+ PRRT group (*n* = 4) received NS injection a day before PTT. On day 0, mice underwent laser irradiation and immediately after they were injected with [^177^Lu]Lu-DOTA-TATE. Tumor growth was followed until tumors reached ~1500 mm^3^. (B) SPECT/CT images 24 h after [^177^Lu]Lu-DOTA-TATE injection for representative PRRT and PTT + PRRT mice. Arrows point to the tumor. (C) Survival curves for all groups. (D–F) Tumor growth curves for all mice in the different groups. Curves plotted until day 50.
