# Supplementary material for: Combination of [177Lu]Lu-DOTA-TATE Targeted Radionuclide Therapy and Photothermal Therapy as a Promising Approach for Cancer Treatment: In Vivo Studies in a Human Xenograft Mouse Model
Source: Pharmaceutics. 2022 Jun 16;14(6):1284. doi: 10.3390/pharmaceutics14061284 (PMC9227845; doi:10.3390/pharmaceutics14061284)
Supplement: Supplementary file 1 [file pharmaceutics-14-01284-s001.zip › pharmaceutics-1745634-supplementary.pdf]

Supplementary material

Combination of [^{177}Lu]Lu-DOTA-TATE Targeted Radionuclide Therapy and Photothermal Therapy as a Promising Approach for Cancer Treatment: In Vivo Studies in a Human Xenograft Mouse Model

Marina Simón [1], Jesper Tranekjær Jørgensen[1], Harshvardhan A. Khare[1], Camilla Christensen[1,2], Carsten Haagen Nielsen[1,2] and Andreas Kjaer [1*]

[1]Department of Clinical Physiology and Nuclear Medicine & Cluster for Molecular Imaging, Copenhagen University Hospital – Rigshospitalet & Department of Biomedical Sciences, University of Copenhagen, 2100 Copenhagen, Denmark

[2]Minerva Imaging, 3650 Ølstykke, Denmark

[*]Correspondence: akjaer@sund.ku.dk

Nanoshell-based photothermal therapy followed by [^{177}Lu]Lu-DOTA-TATE radionuclide therapy for the treatment of NCI-H69 tumor-bearing mice

This initial study was carried out prior to the experiments reported in the main article, with the aim to evaluate if PTT performed before PRRT could improve outcome compared to PRRT as monotherapy.

NCI-H69 tumor-bearing mice were divided into three groups. The first group did not get any treatment (Control group, n = 5). The second group was injected with ~30 MBq of [^{177}Lu]Lu-DOTA-TATE on day 0 (PRRT group, n = 7). The third group was injected with NS on day -1 and underwent a five-minute laser irradiation on day 0, subsequently followed by a ~30 MBq of [^{177}Lu]Lu-DOTA-TATE injection (PTT + PRRT group, n = 4). The study timeline is detailed in Figure S1A. Two mice per group from the PRRT and PTT + PRRT groups were SPECT/CT scanned 24 hours after injection of [^{177}Lu]Lu-DOTA-TATE (Figure S1B). Tumor growth was followed by measuring tumor size with a caliper and mice were euthanized when tumors reached ~1500 mm^3 (Figure S1D-F).

On SPECT/CT scans, a high specific [^{177}Lu]Lu-DOTA-TATE uptake was observed in the tumors of mice receiving PRRT. However, the tumor uptake was noticeably lower in the tumors of the mice that had undergone PTT before [^{177}Lu]Lu-DOTA-TATE injection (PTT + PRRT group), and this correlated with the significantly worse treatment outcome observed for this group (Figure S1C). After an initial tumor-shrinking effect was observed for both groups five to seven days after [^{177}Lu]Lu-DOTA-TATE injection, tumor growth was exacerbated for the PTT + PRRT group when compared to the PRRT group (Figure S1D-E, $p < 0.01$). Median survival was reduced from 48 days for the PRRT group to 31 days for the PTT + PRRT group. The survival for the Control group was 20 days (Figure S1C).

As a result, for all studies going forward, PTT was performed after [^{177}Lu]Lu-DOTA-TATE administration. Additionally, the dose was reduced from 30 MBq to 20 MBq in the main study to enable prompter evaluation of the combination strategy.

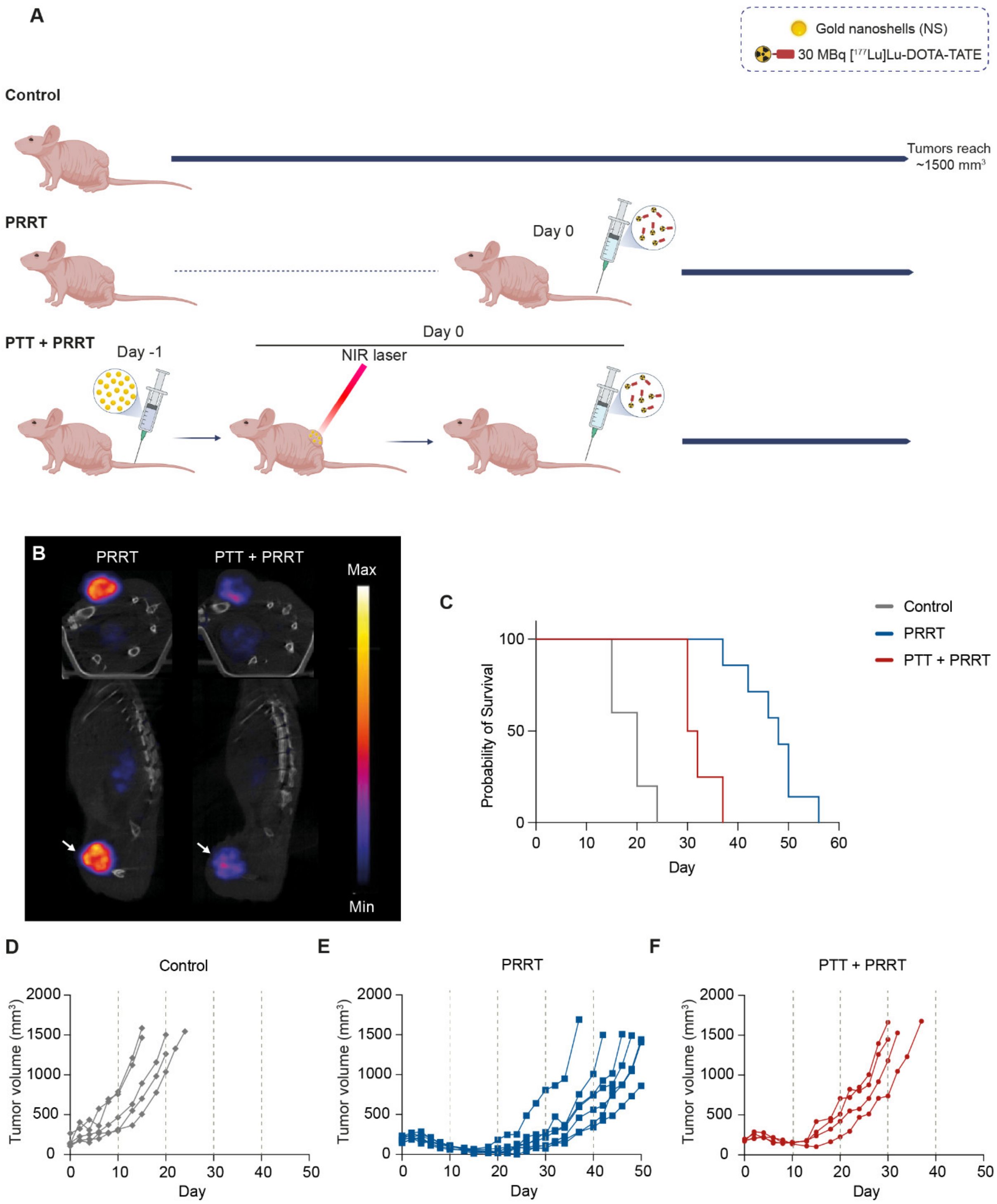

Figure S1: PTT followed by PRRT for the treatment of NCI-H69 tumor-bearing mice. A. Study timeline. The control group (n = 5) did not receive any treatment. The PRRT group (n = 7) was injected with ~30 MBq of [^{177}Lu]Lu-DOTA-TATE on day 0. The PTT+ PRRT group (n = 4) received NS injection a day before PTT. On day 0, mice underwent laser irradiation and immediately after they were injected with [^{177}Lu]Lu-DOTA-TATE. Tumor growth was followed until tumors reached ~1500 mm^3. **B.** SPECT/CT images 24 hours after [^{177}Lu]Lu-DOTA-TATE injection for representative PRRT and PTT + PRRT mice. Arrows point to the tumor. **C.** Survival curves for all groups. **D-F.** Tumor growth curves for all mice in the different groups. Curves plotted until day 50.
